# 3-M syndrome: a growth disorder associated with *IGF2* silencing

**DOI:** 10.1530/EC-13-0065

**Published:** 2013-11-18

**Authors:** P G Murray, D Hanson, T Coulson, A Stevens, A Whatmore, R L Poole, D J Mackay, G C M Black, P E Clayton

**Affiliations:** 1 Centre for Paediatrics and Child HealthInstitute of Human Development, Faculty of Medical and Human Sciences, University of ManchesterManchesterUK; 2 Faculty of MedicineUniversity of SouthamptonSouthamptonUK; 3 Centre for Genetic Medicine, Institute of Human DevelopmentFaculty of Medical and Human Sciences, University of ManchesterManchesterUK; 4 5th Floor Research, Royal Manchester Children's HospitalCentral Manchester University Hospitals NHS Foundation Trust, Manchester Academic Health Sciences CentreOxford Road, Manchester, M13 9WLUK; 5 Genetic Medicine, St Mary's HospitalCentral Manchester University Hospitals NHS Foundation Trust, Manchester Academic Health Sciences CentreOxford Road, Manchester, M13 9WLUK

**Keywords:** SGA, IGF2, 3-M syndrome, CUL7, OBSL1, CCDC8

## Abstract

3-M syndrome is an autosomal recessive disorder characterised by pre- and post-natal growth restriction, facial dysmorphism, normal intelligence and radiological features (slender long bones and tall vertebral bodies). It is known to be caused by mutations in the genes encoding cullin 7, obscurin-like 1 and coiled-coil domain containing 8. The mechanisms through which mutations in these genes impair growth are unclear. The aim of this study was to identify novel pathways involved in the growth impairment in 3-M syndrome. RNA was extracted from fibroblast cell lines derived from four 3-M syndrome patients and three control subjects, hybridised to Affymetrix HU 133 plus 2.0 arrays with quantitative real-time PCR used to confirm changes found on microarray. IGF-II protein levels in conditioned cell culture media were measured by ELISA. Of the top 10 downregulated probesets, three represented *IGF2* while *H19* was identified as the 23rd most upregulated probeset. QRT-PCR confirmed upregulation of *H19* (*P*<0.001) and downregulation of *IGF2* (*P*<0.001). Levels of IGF-II secreted into conditioned cell culture medium were higher for control fibroblasts than those for 3-M fibroblasts (10.2±2.9 vs 0.6±0.9 ng/ml, *P*<0.01). 3-M syndrome is associated with a gene expression profile of reduced *IGF2* expression and increased *H19* expression similar to that found in Silver–Russell syndrome. Loss of autocrine IGF-II in the growth plate may be associated with the short stature seen in children with 3-M syndrome.

## Introduction

3-M syndrome (named after the first three authors to describe the condition) is an autosomal recessive disorder characterised by impaired pre- and post-natal growth, facial dysmorphism (triangular shaped face, anteverted nares, full fleshy lips), prominent heels, normal intelligence and, in some, radiological features (slender long bones and tall vertebral bodies). It is caused by loss of function mutations in the genes encoding cullin 7 (*CUL7*) (1), obscurin-like 1 (*OBSL1*) (2) and coiled-coil domain containing 8 (*CCDC8*) (3). CUL7 is a scaffold protein forming part of an E3 ubiquitin ligase enzyme responsible for cytoplasmic protein degradation (4), while OBSL1 is a cytoskeletal adaptor protein which localises to the perinuclear region (5). The function of CCDC8 is unknown, but it binds to OBSL1 (3) and is required for p53-mediated apoptosis (6).

The mechanisms leading to the growth impairment seen in 3-M syndrome remain unclear, but are likely to relate to abnormalities in basic cellular growth as well as alterations in cellular responses to growth factor stimulation. The *Cul7*^*−/−*^ mouse displays impaired pre-natal growth and abnormalities in placental vasculature, but dies from respiratory distress after birth (7). Suggested targets for the CUL7 containing E3 ubiquitin ligase enzyme include cyclin D1 (8) and IRS1 (9). Altered IGF-I signalling with increased activation of the downstream signalling molecule AKT was identified in *Cul7*^*−/−*^ mouse embryonic fibroblasts (MEFs) (9), associated with poor cell growth and senescence. Overexpression of CUL7 in an immortalised cancer cell line leads to decreased p53-mediated apoptosis (10, 11, 12). In contrast to the data in MEFs, AKT signalling was reduced in human skin fibroblast cell lines derived from 3-M syndrome patients (13) (including one patient with a *CUL7* nonsense mutation). Alterations in the levels of the insulin-like growth factor-binding proteins (IGFBPs) have been identified in 3-M syndrome patient cell lines, both at the RNA level for IGFBP2 and 5 (14) and at the protein level for IGFBP2, 5 and 7 (13). Alterations in IGFBP levels and IGF-I signal transduction are seen in cell lines with *OBSL1* and *CCDC8* mutations (13) as well as *CUL7* mutations; there is, however, a paucity of other data on the link between *OBSL1*, *CCDC8* and *CUL7* and the mechanism of growth impairment.

Although 3-M syndrome is considered to be a relatively uncommon disorder, it is probably an under recognised condition (6); its core characteristics of pre- and post-natal growth impairment are shared with all small for gestational age (SGA) children with failure of catch up growth. This includes many children in whom there is as yet no clear mechanism of growth impairment. The aim of this study was to identify novel potential mechanisms of growth impairment in 3-M syndrome, as an exemplar condition for SGA, by examining the transcriptome of skin fibroblast cell lines derived from 3-M patients. Skin fibroblast cell lines have previously been useful in the study of other growth disorders (15, 16). An understanding of the mechanisms of growth impairment in 3-M syndrome could lead to insights into the causation of poor growth in other SGA children and potential targets for molecular diagnostics.

## Subjects and methods

### Patients

Skin fibroblast cell lines were derived from four 3-M syndrome patients and three control subjects. Biopsies were obtained from the forearm after application of EMLA cream (AstraZeneca). The patients included one male with a homozygous *CUL7* mutation (c.4191delC p.H1379HfsX11), one male with a homozygous *OBSL1* mutation (c.1273insA, p.T425NfsX40, referred to as OBSL1M here), one female with a homozygous *OBSL1* mutation (c.1273insA, p.T425NfsX40, referred to as OBSL1F) and one female with a homozygous *CCDC8* mutation (c.84dup, p.L29X). The three control fibroblast cell lines (two males and one female) were derived from skin obtained during removal of skin tags. All patients and control subjects were prepubertal at the time the skin samples were obtained. All patients with 3-M syndrome had clinical features of the condition including growth impairment.

### Cell culture

Fibroblast cells were cultured in 75 cm^2^ cell culture flasks (Corning, Tewkesbury, MA, USA) in DMEM (Invitrogen Paisley, Renfrewshire, UK) supplemented to a final concentration with 10% foetal bovine serum (Invitrogen), 50 units/ml penicillin, 50 μg/ml streptomycin, 2 mM glutamine and 2.5 μg/ml amphoteracin B (Invitrogen).

### WST-8 cell growth assay

Cells were seeded at a density of 1000 cells/cm^2^ in 96-well cell culture plates (Corning) in 100 μl cell culture media: 24 and 72 h after seeding, 10 μl WST-8 was added to each well, the plate was incubated for 2 h at 37 °C before measuring absorbance at 450 nm on a u.v. spectrophotometer (Bio-Rad Benchmark microplate reader, Bio-Rad UK). For each cell line at each time measurement, a minimum of eight independent wells were examined on three separate occasions.

### 5-Ethynyl-2′-deoxyuridine incorporation

Cells were seeded at a density of 1000 cells/cm^2^ into 8-well chamberslides (Scientific Laboratory Supplies, Hessle, Yorkshire, UK) and incubated for 24 h in 600 μl cell culture media at 37 °C in 5% CO_2_. After 24 h, the culture medium was removed and replaced with media containing 40 μM 5-ethynyl-2′-deoxyuridine (EdU) for 3 h with the cells incubated in standard conditions. The media was then removed and the cells washed, fixed and permeabilised. EdU incorporation was assessed using the Click-iT EdU Alexa Fluor 488 Imaging Kit *for 50 coverslips (Invitrogen) as per the manufacturer's instructions. DAPI was used to identify the total number of cells present. Three independent fields containing at least 50 cells per field were examined for each cell line and the experiment was repeated on three occasions. A Leica CTR 5000 microscope was used to visualise the cells incorporating EdU.

### Cleaved caspase-3 ELISA

Cleaved caspase-3 was measured with the PathScan Cleaved Caspase-3 ELISA (New England Biolabs, Hitchin, Hertfordshire, UK). Cells were seeded in 6-well plates at 1000 cells/cm^2^ for each cell line: 48 h after seeding, the media were removed and cell lysate was generated as per the manufacturer's instructions. Absorbance at 450 nm was measured on a u.v. spectrophotometer (Bio-Rad Benchmark microplate reader, Bio-Rad UK).

### RNA extraction and transcriptome analysis

RNA was extracted from the fibroblasts using the RNAEasy kit (Qiagen, Manchester, UK) as per the manufacturer's instructions and supplied to the University of Manchester Microarray Facility (Faculty of Life Sciences, University of Manchester, UK). RNA quality was assessed using an Agilent 2100 Bioanalyser: 500 ng of total RNA per cell line was reverse transcribed using a T7 Oligo dT primer. An *in vitro* transcription reaction was used to generate biotinylated cRNA, which was purified, fragmented and hybridised to an Affymetrix HU-133 Plus 2.0 chip (Affymetrix, Santa Clara, CA, USA).

Microarray data were analysed using Propagating Uncertainty Microarray Analysis (PUMA – http://www.bioinf.manchester.ac.uk/resources/puma/). This process obtains a value for expression for each probeset on the microarray chip and involves normalising gene expression both within and between chips. Probesets were defined as being up- or downregulated if there was a ±1.5-fold difference in the expression between the control and 3-M samples with an expression level >50 (arbitrary units) in at least one cell line.

PUMA was also used to undertake principle component analysis (PCA) with probability of positive log-ratio (PPLR) to examine any differences in gene expression between control and 3-M fibroblasts. PPLR values closer to +1 indicate those probesets that are most likely to be upregulated and values closer to −1 indicate those most likely to be downregulated. In addition to PCA, quality control of the arrays was assessed with dCHIP (http://biosun1.harvard.edu/complab/dchip/).

Gene ontology and pathway analysis were performed with the use of the National Institute's Health Database for Annotation, Visualisation, Integrated Discovery (NIH DAVID) (http://david.abcc.ncifcrf.gov/).

### Quantitative PCR

One microgram RNA (derived independently from the samples used for microarray) was reverse transcribed using the high capacity RNA to cDNA kit (Applied Biosystems). *IGF2* and *H19* mRNA levels were assessed using TaqMan assays (Hs01005963_m1 and Hs00262142_g1) with a cyclophyllin A probe (4333763T) for control gene expression. The other genes were assayed using SYBR green with GAPDH as the control gene (primer sequences available on request). Relative fold gene expression for the target gene in the 3-M cell lines was calculated as 2^−^^ΔΔ^^*C*T^. For each experiment, three independent RNA extractions were assayed with three technical replicates.

### Methylation analysis of H19 and IGF2

One microgram peripheral leukocyte or fibroblast-derived genomic DNA was treated with bisulphite using the EZ-DNA Methylation kit, according to manufacturer's instructions (Zymo Research, Orange, CA, USA), except that DNA was eluted in 50 μl. Methylation-specific PCRs were performed in duplicate within the H19 promoter and IGF2 DMR0 (as described in Poole *et al*. (17)) and the products were visualised by capillary electrophoresis on an ABI 3130 Genetic Analyzer (Applied Biosystems). Peak height ratiometry was performed and normalised to control samples.

Pyrosequencing was performed in duplicate, interrogating both the H19 ICR (as described in Poole *et al*. (17)) and IGF2P0 DMR0 (as described in Murrell *et al*. (18)). Primer sequences for all assays are provided in Tables 1 and 2.

### Measurement of IGF-II in cell culture medium

Conditioned cell culture medium was obtained by incubating serum-free media with the relevant skin fibroblast cell line for 7 days. IGF-II levels were measured using an Active Non-Extraction IGF-II ELISA (Beckman Coulter, High Wycombe, Buckinghamshire, UK). IGF-II concentrations are much lower in cell culture medium than serum (for which the kit was designed) and this required some amendment to the standard kit protocol. Up to 400 μl conditioned cell culture medium was added to 550 μl sample buffer 1 and incubated at room temperature for 30 min: 950 μl sample buffer 2 was then added and the tube was vortexted. Fifty microlitre of the treated samples were added to each well of the ELISA plate and the remainder of the process was performed as per the manufacturer's instructions. Total protein concentration was measured in the conditioned media using Bio-Rad protein assay dye reagent (Bio-Rad) as per the manufacturer's instructions. Protein concentration of the control media was normalised to 1 and the IGF-II concentration adjusted for the total protein concentration in each cell line's conditioned media.

## Results

### Whole transcriptome analysis

**Figure 1:**
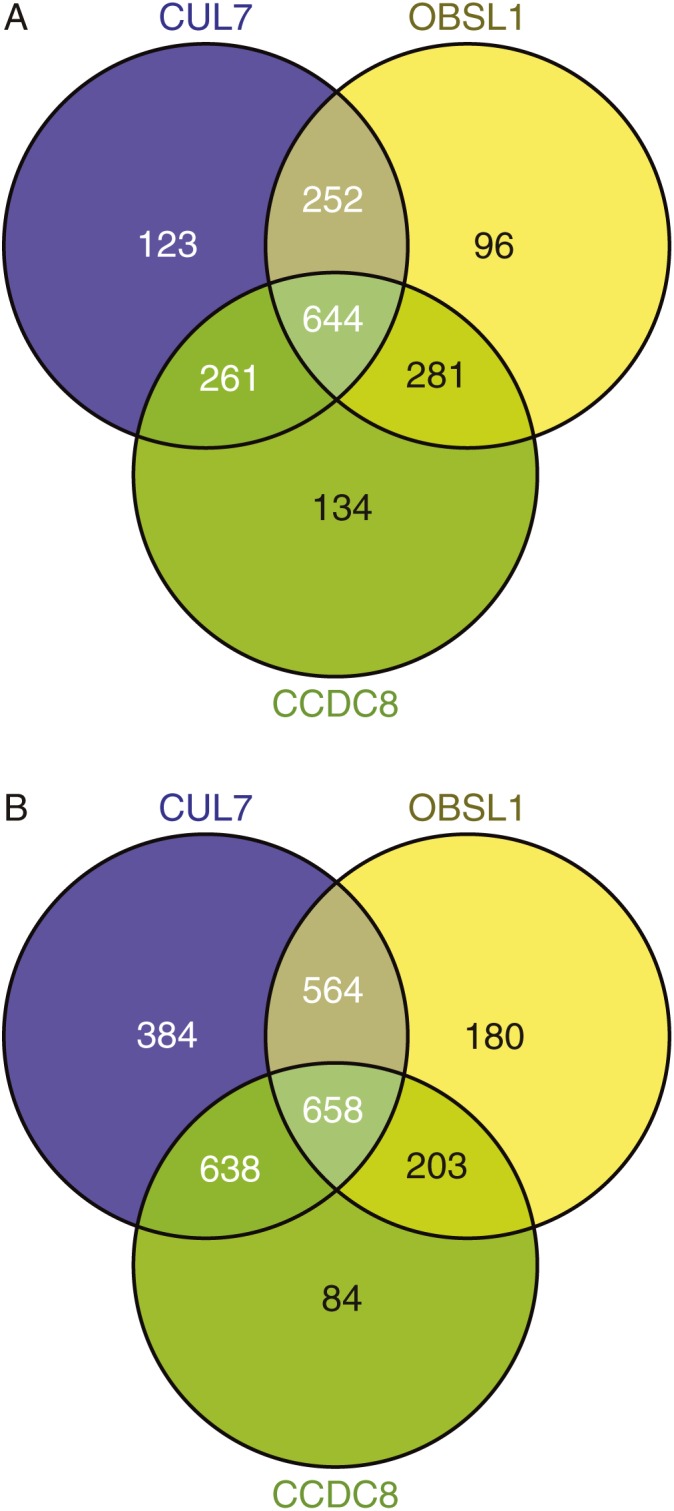
Venn diagrams of (A) up- and (B) downregulated probesets with expression level >50 and fold change (FC) >±1.5 compared to control.

There were 644 probesets identified as being upregulated and 658 identified as being downregulated in all three groups of 3-M patients compared with controls (Fig. 1). The top 10 up- and downregulated probesets are listed in Tables 3 and 4 respectively. Table 5 lists the top 20 up- and downregulated probesets for each cell line (*CUL7*, *OBSL1* or *CCDC8* mutation) and indicates which of these probesets are shared between more than one group. The majority of probesets is shared between more than one group and this suggests that there is a common set of genes dysregulated in 3-M syndrome.

Among the upregulated probesets were two homeobox genes, *HOXC6*, a transcription factor expressed in the developing skeleton (19), and *HOXA9*, a transcription factor involved in myeloid differentiation linked with increased cell proliferation in leukaemia (20). Other upregulated genes included *GPC6* (loss of function mutations result in the short stature condition omodysplasia (21)) as well as zinc finger protein of cerebellum 1 (*ZIC1*) and *PCP4* both of which are known to be differentially expressed in tumours (22, 23). Three out of the top 10 downregulated probesets represented *IGF2* which encodes a 7.5 kDa secreted hormone known to be a regulator of intra-uterine growth (24).

Q-PCR validation of a six up-and downregulated genes was undertaken (Table 6). In all cases, the Q-PCR result confirmed the findings on the microarray.

Gene ontology analysis of the top 500 up- and downregulated probesets comparing all four 3-M cell lines with controls identified terms including skeletal system morphogenesis, cell adhesion and cell–cell signalling as being over represented (Benjamini–Hochberg adjusted *P* value <0.05). Cellular compartment terms significantly over-represented all related to the extra-cellular region.

Of the differentially regulated genes identified, the gene most closely linked with impaired growth was *IGF2*. Hypomethylation of the H19 differentially methylated region leads to *IGF2* silencing in the Silver–Russell syndrome (SRS) (25). SRS shares key features with 3-M syndrome, namely the pre- and post-natal growth restriction with normal head size and triangular facies. It was therefore decided to focus further studies on *IGF2*.

### *IGF2* expression and protein concentrations in conditioned cell culture media

**Figure 2:**
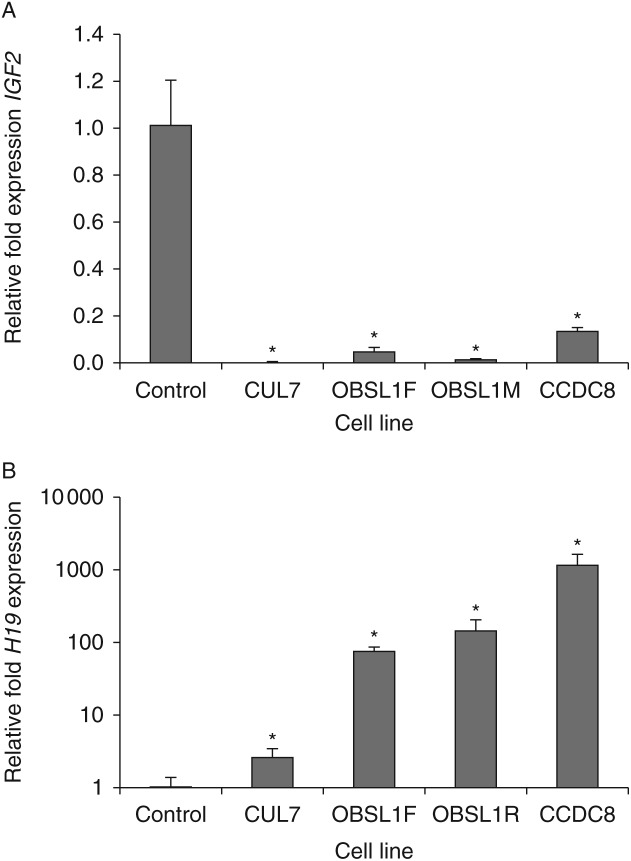
Relative fold expression of (A) *IGF2* and (B) *H19* measured using quantitative PCR in all 3-M cell lines. Expression of *IGF2* is reduced while *H19* is increased. *P<0.05.

Q-PCR using three independently extracted RNA samples (each sample run in triplicate) confirmed the decrease in *IGF2* expression with relative fold expression of 0.0019±0.0009 for CUL7 (*P*<0.001), 0.0155±0.0021 for OBSL1M (*P*<0.001), 0.0497±0.0170 for OBSL1F (*P*<0.001) and 0.1355±0.0146 for CCDC8 (*P*<0.001) compared with controls (Fig. 2).

Although not present in the top 10 upregulated probesets, the H19 non-coding RNA was represented by the 23rd most upregulated probeset (FC 38, PPLR 1). Q-PCR of H19 confirmed that it was upregulated in all four 3-M cell lines (Fig. 2). Relative fold expression was 2.5±0.8 for CUL7 (*P*<0.001), 140±53 for OBSL1M (*P*<0.001), 72±12 for OBSL1F (*P*<0.001) and 1106±435 for CCDC8 (*P*<0.001).

**Figure 3:**
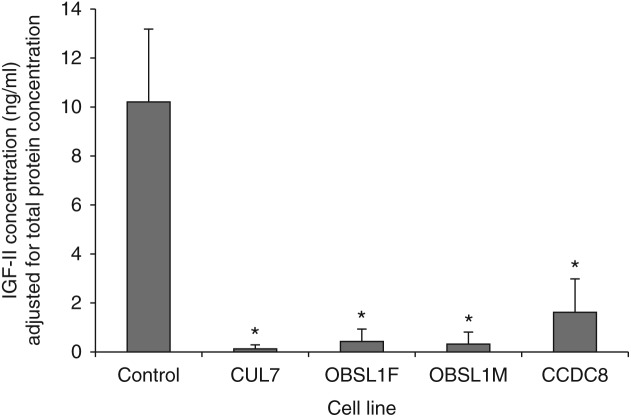
IGF-II concentrations in conditioned cell culture media are significantly reduced for all 3-M syndrome fibroblast cell lines. *P<0.05 compared to control. IGF-II concentrations are adjusted for total protein concentration in the cell culture media.

Concentrations of IGF-II were reduced in conditioned cell culture media from all four 3-M cell lines compared with control cell lines (Fig. 3). The mean IGF-II concentration for the three control cell lines after adjustment for total protein concentration in the media was 10.2±2.9 ng/ml, compared with 0.1±0.2 ng/ml for the CUL7 cell line (*P*<0.001), 0.3±0.4 ng/ml for the OBSL1M cell line (*P*<0.001), 0.4±0.5 ng/ml for the OBSL1F cell line (*P*<0.001) and 1.6±1.3 ng/ml for the CCDC8 cell line (*P*<0.001).

Overexpression of *H19* and silencing of *IGF*2 in SRS are caused by changes in methylation in the H19 differentially methylated region. Methylation-specific PCR and pyrosequencing of the *H19* ICR, *H19* promotor and *IGF2* DMR0 identified no differences in methylation between control and 3-M syndrome subjects, for both peripheral leucocyte and fibroblast-derived DNA.

### Cell proliferation and apoptosis

**Figure 4:**
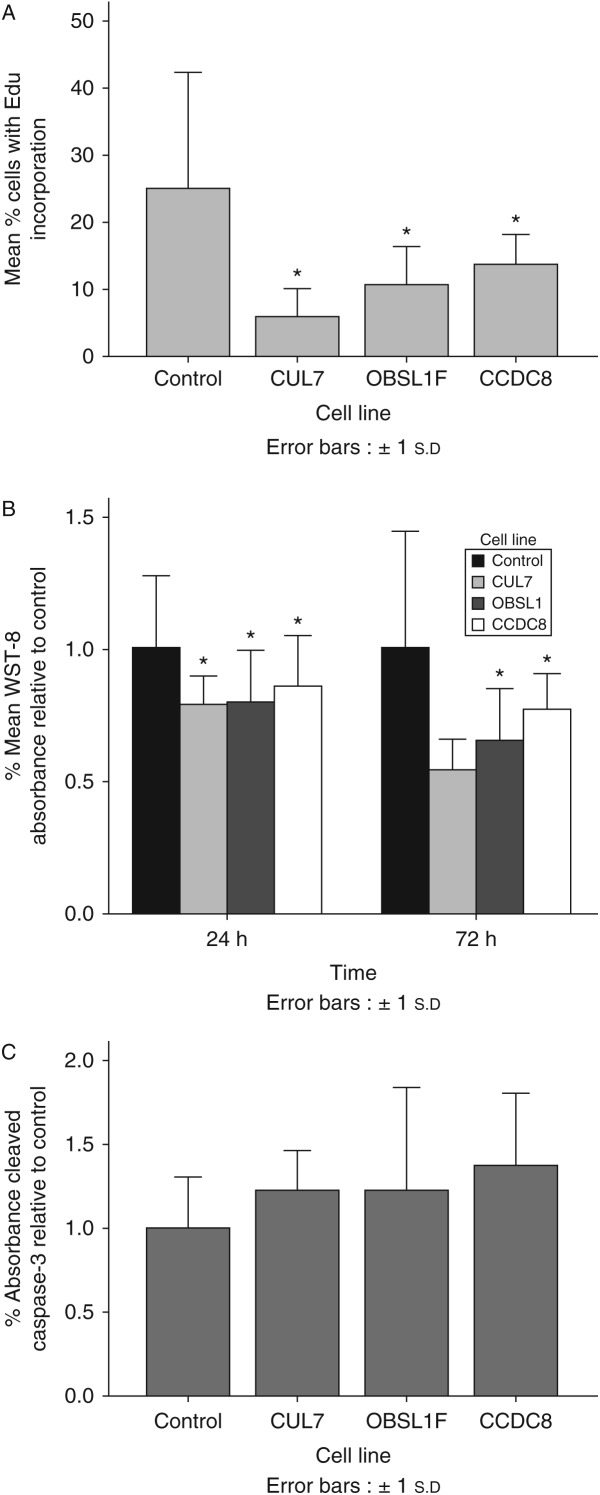
Reduced cell proliferation with no increase in apoptosis is seen in fibroblasts from 3-M syndrome patients. (A) Decreased incorporation of EdU measured at 48 h. (B) Decreased cell proliferation at 24 and 72 h after seeding measured by WST-8. (C) No difference in apoptosis as measured by Cleaved Caspase-3 ELISA. *P<0.05.

Cell proliferation was assessed via incorporation of EdU and by a WST-8 assay. Incorporation of EdU 48 h after its addition to cell culture media was reduced for all 3-M fibroblast cell lines compared with control (Fig. 4A, *P*<0.05), while cell proliferation as measured by colorimetric change induced by WST-8 was reduced at 48 and 72 h after seeding for 3-M fibroblast cell lines compared with control (Fig. 4B, *P*<0.05). Cleaved caspase-3, a biomarker of apoptosis was not significantly different between control and 3-M fibroblasts (Fig. 4C).

## Discussion

The aim of this study was to identify novel pathogenic mechanisms underlying the growth failure of patients with 3-M syndrome, which could potentially be relevant to other patients born SGA with the failure of post-natal growth but no defined aetiology. Previous work has examined the role of CUL7 and OBSL1 either in mouse studies or using gene overexpression or knockdown strategies in immortalised cancer cell lines. The limitations of mouse studies are clear from the death of the mice in the neonatal period (a feature not commonly seen in humans with 3-M syndrome). The mouse thus gives no opportunity to study the effects on post-natal growth and also indicates significant differences in the result of loss of CUL7 between species. Studies using temporary over/under expression strategies in immortalised cells yield useful data, but the extrapolation from these findings to normal human growth is not clear. This study therefore used patient-derived fibroblast cell lines.

It is clear that there is a common set of genes dysregulated in 3-M syndrome. The top upregulated gene was *Zic1*, a transcription factor which, in mouse, is predominantly expressed within the nervous system with the highest levels of expression in the cerebellum (26). *ZIC1* expression is downregulated in gastric carcinomas (23) and increased in desmoid tumour fibroblasts (27) and brain tumours (medulloblastomas and meningiomas) (28, 29). Several other genes in the top 10 upregulated probesets are also overexpressed in tumours including *PCP4* in leiomyomas (22), *HOXC6* in oesophageal (30), breast (31) and lung carcinomas (32) and *IL16RA2* in glioblastomas (33), prostate cancer and adrenocortical tumours (34). This indicates that their overexpression in 3-M fibroblasts could be an attempt to increase cell proliferation. Data on siRNA-mediated knockdown and overexpression of *HOXC6* in a gastric carcinoid cell line are consistent with this hypothesis as overexpression leads to improved growth while loss of *HOXC6* leads to impaired cell growth (35).

Glypican 6 (*GPC6*), in the top 10 downregulated probesets, is a heparan sulphate proteoglycan, which is linked to the extracellular surface of the cell membrane. Glypicans are expressed during development and are thought to control availability of local growth factors (36). Loss of function mutations in GPC6 lead to impaired endochondral ossification and cause the short stature condition omodysplasia (21). Loss of function mutations in glypican 3 (GPC3) causes the overgrowth disorder Simpson–Golabi–Behmel syndrome (SGBS). GPC3 interacts with IGF-II and it was initially hypothesised that GPC3 binds to and sequesters IGF-II; thus the overgrowth in SGBS is caused by increased availability of IGF-II (37). More recent data on mouse indicate that the overgrowth of *Gpc3* null mice is independent of IGF-II (38), while data on the role of GPC3 in the growth of cancer cell lines are inconsistent with some studies suggesting that GPC3 suppressed growth in an IGF-dependant manner (39) while others identified GPC3 as a growth-promoting protein (40, 41). Of note, of the two probesets in our microarray designed to detect the expression of GPC3, one did not detect expression of GPC3 (defined in this study as an expression level >50) in any cell line while the other probeset identified a modest downregulation (FC −1.76 PPLR 0.44).

Three of the top 10 downregulated probesets represented *IGF2* with the smallest fold change being −157. The second most downregulated probeset represented leptin, a 16 kDa adipocyte-derived hormone which plays a central role in the regulation of body weight, both by inhibiting food intake and increasing energy expenditure (42, 43). Downregulation of leptin in 3-M syndrome may represent a response to the patients slim body habitus or be a signal to drive energy intake in order to promote growth. *BEX1*, *PTGDS*, *GRIK2* and *WFDC1*, also in the top 10 downregulated probesets, have all been identified as downregulated in tumours (44, 45) or as inhibitors of cell proliferation in immortalised cell lines (46, 47, 48). Thus in common with several of the genes identified as being upregulated these changes are likely to represent a response to increase cell proliferation.

While many of the identified changes in gene expression, such as the overexpression of GPC6, are likely to be a compensatory response to the growth impairment, we hypothesised that there would be a smaller number of genes with altered expression which are key to the pathogenic process underlying 3-M syndrome. The most obvious of such candidate was *IGF2*, which was represented by three of the top 10 downregulated probesets. The encoded protein IGF-II is a 7.5 kDa secreted hormone that acts to increase cell proliferation via stimulation of the IGF1R. It is widely expressed during the development and is a major regulator of intra-uterine growth. *IGF2* expression is regulated by methylation of the H19 region (H19 DMR); hypomethylation of the H19 DMR with subsequent *IGF2* gene silencing leads to the short stature condition SRS (25). SRS shares several key features with 3-M syndrome: intra-uterine growth retardation, post-natal growth impairment, relatively normal head size, normal intelligence and a triangular shaped face. Other downregulated genes identified which could potentially be implicated in the pathogenesis of 3-M syndrome included *LGR5* (a member of the G-protein-coupled receptor superfamily) and *COL4A1* (the main component of type IV collagen which forms basement membrane); silencing of expression of these genes is associated with decreased cell proliferation (49) while in tumours (50, 51), they have been found to be upregulated. Of the top 10 upregulated probesets, the only gene identified as potentially being involved in the pathogenesis of 3-M syndrome was COL14A1, a large glycoprotein of the extracellular matrix which has an anti-proliferative effect on fibroblasts (52) and knockdown in renal cancer cells, which results in increased growth (53).

Given the findings of reduced IGF-II production from 3-M syndrome fibroblasts, it is likely that local production of IGF-II is reduced with loss of its autocrine/paracrine effects. Loss of local IGF-II in the growth plates and other tissues leads to growth impairment both pre- and post-natally. The reduced cell proliferation with no change found in a biomarker of apoptosis would be consistent with a reduction in the presence of a growth factor. While there is significant phenotypic overlap between 3-M syndrome and SRS, there are also phenotypic differences. These are likely to be due to additional functions of the proteins affected in 3-M syndrome.

The mechanisms through which *IGF2* expression is reduced in 3-M syndrome remain unclear. It does not appear to be via the same mechanism as is found in SRS, i.e. hypomethylation at H19 DMR. It is possible that there may be an epigenetic change which has not been recognised but this appears unlikely. There may be another mechanism, such as alteration in CCCTC-binding factor concentrations or activity that could lead to the same gene expression pattern.

Height at presentation in 3-M syndrome is lowest in patients with *CUL7* mutations and highest in those with *CCDC8* mutations (13). Of interest, the pattern of *IGF2* expression and IGF-II production mirrored the growth phenotype of the patients with the lowest IGF-II production in the CUL7 cell line and the highest IGF-II production in the CCDC8 cell line.

In conclusion, this study demonstrates that there is reduced expression of *IGF2* in 3-M syndrome linking the pathogenesis to that of SRS. The mechanisms underlying the silencing of *IGF2* in 3-M syndrome are unclear, but do not appear to involve hypomethylation at the H19 DMR.

## Figures and Tables

**Table 1:** Primer sequences for methylation-specific PCR.

**DMR**	**Chr**	**Chromosomal location GRCh37**	**Methylated allele size**	**Unmethylated allele size**	**Methylated primer**	**Unmethylated primer**	**Universal FAM-labelled primer**
H19 promotor	11p15	chr11: 2 019 455–2 019 764	pat 295	mat 305	CGTTTGTTAGTAGAGTGCGTTCGCGAGTCG	GGTTGTTTATTGTTTGTTAGTAGAGTGTGTTTGTG	ATAACAGAAAAAACCCCTTCCTACCACCATCAC
IGF2P0	11p15	chr11: 2 169 485–2 169 651	pat 155	mat 163	GTTTGACGAGGTTAGTGAGGGACGGCG	ATAGTTTTGTTTGAtGAGGTTAGTGAGGGATGGTG	CCAAAACAATTTCCCTAAAAATACTCATTCATAC

**Table 2:** Primer sequences for pyrosequencing.

**DMR**	**Chr**	**Chromosomal location GRCh37**	**Methylated allele size**	**Unmethylated allele size**	**Primer 1**	**Primer 2 (biotinylated)**	**Sequencing primer(s)**
IGF2 DMR0	11p15	chr11: 2 169 328–2 169 582	NA	NA	TGAGGATGGGTTTTTGTTTGGTAT	TCCTCAATCCACCCAAAATAATAT	AAAAGTTATTGGATATATAGT or GGGGTGGAGGGTGTA
H19 ICR1	11p15	chr11: 1 977 650–1 977 877	NA	NA	GTATAGTATATGGGTATTTTTGGAGG	CCATAAATATCCTATTCCCAAATAACC	GTTTYGGGTTATTTAAGTT

**Table 3:** Top 10 upregulated probesets comparing all 3-M cell lines (*n*=4) with control (*n*=3).

**Gene title**	**Gene symbol**	**Mean expression level control**	**Mean expression level 3-M**	**Fold difference 3-M/control**	**PPLR**
Zic family member 1	*ZIC1*	0.16	171.85	1087.41	1.00
Purkinje cell protein 4	*PCP4*	0.36	185.70	513.42	1.00
Homeobox C6	*HOXC6*	2.40	615.78	256.07	0.99
Homeobox A10	*HOXA10*	1.40	311.76	223.27	0.55
Homeobox A9	*HOXA9*	1.83	346.95	189.98	1.00
Interleukin 13 receptor, alpha 2	*IL13RA2*	6.27	779.20	124.27	1.00
Collagen, type XIV, alpha 1	*COL14A1*	1.28	145.45	113.88	1.00
Glypican 6	*GPC6*	5.51	591.28	107.25	1.00
Clusterin	*CLU*	7.84	795.86	101.45	1.00
Solute carrier member 15	*SLC6A15*	1.42	117.15	82.29	1.00

PPLR, probability of positive log-ratio.

**Table 4:** Top 10 downregulated probesets comparing all 3-M cell lines (*n*=4) with control (*n*=3).

**Gene title**	**Gene symbol**	**Mean expression level control**	**Mean expression level 3-M**	**Fold difference 3-M/control**	**PPLR**
Insulin-like growth factor 2	*IGF2*	2118.84	0.06	−38 253.37	0.00
Leptin	*LEP*	64.69	0.10	−642.51	0.00
Insulin-like growth factor 2	*IGF2*	94.15	0.17	−549.78	0.00
Brain expressed, X-linked 1	*BEX1*	369.40	1.30	−283.28	0.00
Prostaglandin D2 synthase 21 kDa (brain)	*PTGDS*	136.49	0.62	−219.06	0.00
Collagen, type IV, alpha 1	*COL4A1*	199.37	1.09	−183.08	0.03
Leucine-rich repeat-containing G protein-coupled receptor 5	*LGR5*	270.20	1.51	−179.24	0.01
Insulin-like growth factor 2	*IGF2*	44.95	0.29	−157.09	0.00
Glutamate receptor, ionotropic, kainate 2	*GRIK2*	44.54	0.35	−126.79	0.00
WAP four-disulfide core domain 1	*WFDC1*	37.84	0.37	−101.50	0.00

PPLR, probability of positive log-ratio.

**Table 5:** Top 20 up- and downregulated probesets in the 3-M group as a whole and in each cell line by mutation.

**Up-regulated probesets**				**Down-regulated probesets**			
3-M	CUL7	OBSL1	CCDC8	3-M	CUL7	OBSL1	CCDC8
ZIC1^a^	PCP4^a^	ZIC1^a^	XIST	IGF2^a^	IGF2^a^	IGF2^a^	IGF2^a^
PCP4^a^	ZIC1^a^	PCP4^a^	ZIC1^a^	LEP^a^	LGR5^b^	LEP^a^	CADM1
HOXC6^a^	HOXA10^a^	HOXC6^a^	HOXC6^a^	IGF2^a^	COL4A1^b^	EDIL3^c^	PSG2
HOXA10^a^	COL14A1^b^	COL14A1^b^	HOXA10^a^	–	SFRP2	IGF2^a^	IGF2^a^
HOXA9^a^	HOXA9^a^	SNCA^c^	HOXA9^a^	BEX1^a^	LEP^a^	–	DDX3Y
IL13RA2^a^	IL13RA2^a^	HOXA10^a^	PCP4^a^	PTGDS^b^	DIO2	COL4A1^b^	LGR5
COL14A1^b^	THBS4	HOXA9^a^	PAX6	COL4A1^b^	APOE^c^	BEX1^a^	PTGDS
GPC6^a^	–	CLU^b^	EMCN	LGR5^b^	RARRES2^b^	PTGDS^b^	PSG3
CLU^b^	HOXA11^b^	–	XIST	IGF2^a^	NID2	IGF2^a^	BEX1^a^
SLC6A15^b^	HOXC6^a^	CYP3A5	PAX6	GRIK2^c^	TFAP2A^b^	HAPLN1	RPS4Y1
HOXA10^a^	TNXB	SLC6A15^b^	GPC6^a^	WFDC1^c^	–	IGFBP5	MAOA
HOXA11^b^	KCNB1	IL13RA2^a^	CLU^b^	TFAP2A^b^	LXN	RARRES2^b^	GRIK2
–	WIF1	GPC6^a^	IL13RA2^a^	RARRES2^b^	IGF2^a^	WFDC1^c^	TFAP2A^b^
CLU^b^	GPC6^a^	CLU^b^	HOXA10^a^	APOE^c^	TLR4^a^	HAPLN1	LEP^a^
–	HOXA10^a^	SPON1^c^	XIST	WNT5A^c^	APOE^c^	SYNPO2^c^	IGF2^a^
ABCA6^b^	ST8SIA1	SCARA3^c^	WISP1	EDIL3^c^	ITIH5^c^	WNT5A^c^	TRPC6
SCARA3^c^	ACE	HOXA10^a^	THBD	SIM2	PMEPA1	IGFBP7	FAM19A5
HOXA9^a^	SLC6A15^b^	ABCA6^b^	THBD	–	PSG7	TPD52L1	ITIH5^c^
SPON1^c^	COL14A1^b^	SPON1^c^	H19	SYNPO2^c^	DIO2	MRVI1	ITIH5^c^
TBX5	ABCA6^b^	SNX10	SNCA^c^	PPP1R14A	BEX1^a^	STXBP6	USP9Y

^a^Probesets present in all groups; ^b^probesets present in the 3-M group and two mutation groups; ^c^probesets present in the 3-M group and one mutation group. –, probeset is designed to a gene which was not named at the time of the study.

**Table 6:** Additional validation of gene expression data. Expression of genes identified as being up- or downregulated in the microarray were assessed with Q-PCR. Relative fold expression for each of the seven genes analysed is given for each of the four 3-M cell lines. Expression was normalised to GAPDH and mean control cell line expression.

**Gene**	**CUL7**		**OBSL1 F**		**OBSL1 M**		**CCDC8**	
Relative expression	*P*	Relative expression	*P*	Relative expression	*P*	Relative expression	*P*
*BEX1*	0.06±0.01	<0.001	0.15±0.20	<0.001	0.03±0.02	<0.001	0.20±0.24	<0.001
*LEP*	0.07±0.04	<0.001	0.06±0.03	<0.001	0.01±0.00	<0.001	0.04±0.01	<0.001
*ZIC1*	946±462	<0.001	496±186	0.004	1200±891	<0.001	759±498	0.002
*HOXC6*	31±24	0.006	88±55	0.001	51±38	0.005	100±87	0.009
*HOXA9*	690±586	0.008	501±466	0.012	349±296	0.008	399±342	0.008
*GPC6*	35±6	<0.001	44±10	<0.001	7±2	<0.001	5±1	<0.001
